# Gastroenteritis Outbreak at Holiday Resort, Central Italy

**DOI:** 10.3201/eid1403.070121

**Published:** 2008-03

**Authors:** Giacomo Migliorati, Vincenza Prencipe, Alessandro Ripani, Cristina Di Francesco, Claudia Casaccia, Silvia Crudeli, Nicola Ferri, Armando Giovannini, Maria Maddalena Marconi, Cristina Marfoglia, Valeria Melai, Giovanni Savini, Giampiero Scortichini, Primula Semprini, Franco Maria Ruggeri

**Affiliations:** * Istituto Zooprofilattico Sperimentale dell’Abruzzo and Molise “G. Caporale,” Teramo, Italy; † Istituto Superiore di Sanità, Rome, Italy; ‡ Public Health Department of the Local Health Unit, Teramo, Italy

**Keywords:** Groundwater, drinking water, Campylobacter, infectious gastroenteritis, norovirus, waterborne diseases, rotavirus, salmonella, holiday resort, dispatch

## Abstract

During the summer of 2003, a gastroenteritis outbreak spread throughout a holiday resort in central Italy. Fecally contaminated groundwater and seawater were leaking into the non–drinking-water system, which was found to be connected to the drinking-water system of a large resort. This contamination had a primary role in the onset of the outbreak and spread of the infection.

## Abstract 2

Gastroenteritis Outbreak at Holiday Resort, Central Italy

Gastrointestinal infections are the most common diseases among resort guests (*1*). The role of drinking water in transmission, which involves mostly leakage from non–drinking-water or sewage systems or cross-connection between water supply and wastewater systems (*2*–*4*), has been well documented in the literature.

Several episodes of acute gastroenteritis had been observed in coastal holiday resorts of central Italy before 2003. Our study’s aim was to identify and eliminate the source of infection. The investigation was conducted from June to September 2003 at a resort where the greatest number of cases had been observed. We describe the epidemics, the activities to trace the sources of infection, and the results achieved.

## The Study

**Figure 1:**
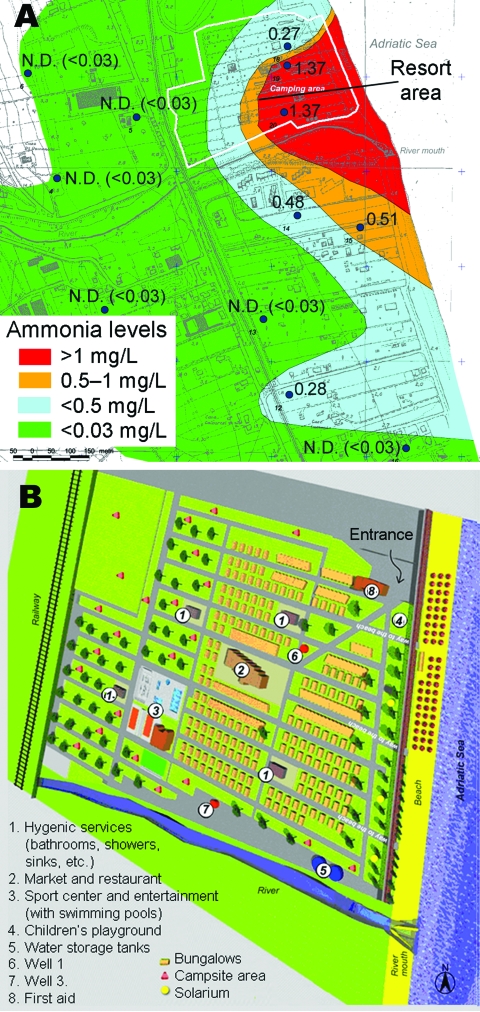
A) Geographic distribution of ammonia residues, central Italy, 2003. Large dots indicate location of wells tested. N.D., not detectable. B) Map of resort area, showing areas of water storage and use.

We conducted a survey at an Italian holiday resort that had a history of gastroenteritis epidemics. The resort can accommodate 4,080 persons and is organized into 2 different areas: 1 for cabins and 1 for campers or tents. Bathrooms, showers, laundry facilities, and a sports center with swimming pools are provided (Figure 1). The last stretch of a small river (the Salinello River) runs on one side of the village.

Our study included an epidemiologic survey (tracing the infection sources of the outbreak) and an environmental survey to check the hypothesized origin of infection. The epidemiologic investigation was a matched case–control study; a case-patient was defined as any person at the resort who reported at least 3 episodes of diarrhea or vomiting within a 24-hour period (*5*). Each case-patient was paired with a randomized control patient of the same age group who had stayed in the resort during the week before the case-patient’s onset of symptoms. Data collected from case-patients and control patients included type of accommodation in the resort, contact with other infected persons, and exposure to possible risk factors in the 3 days before symptom onset. We analyzed selected risk factors with univariate statistical techniques (*6*) and stepwise multivariate logistic regression (*7*).

Stool samples were taken and tested for *Campylobacter* spp., *Escherichia coli* O157, *Salmonella* spp., *Listeria monocytogenes*, *Shigella* spp., *Vibrio cholerae*, *Clostridium perfringens* toxin, *Bacillus cereus* toxin, norovirus (*8*,*9*), and rotavirus (*10*,*11*). For confirmation of norovirus and strain genotyping, samples were further assayed by open reading frame 1 reverse transcription–PCR (RT-PCR) by using biotin-labeled JV12 ad JV13 primers and reverse line blot hybridization (RLBH) (*12*).

**Figure 2:**
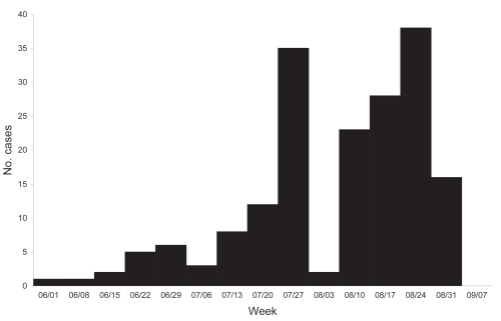
Epidemic curve of cases studied, central Italy, 2003.

We determined that a gastroenteritis epidemic occurred from July 1, 2003, to September 4, 2003 (Figure 2). Overall, 183 case-patients were identified (169 guests and 14 resort employees); 123 (67%) belonged to 2–4 case-family clusters. Approximately two thirds of case-patients had symptoms within 6 days of arrival. We selected and interviewed 181 controls (128 guests and 53 resort employees) for the study.

Sea bathing, use of cabin and shared toilets, and showers supplied with non–drinking water were significantly associated with the disease, as shown in Table 1. Stool samples were collected from 19 patients from July 15, 2003, through September 4, 2003, mainly at the beginning of the epidemic. Of these, 13 samples (68%) were positive for norovirus (2 by PCR, 6 by ELISA, and 5 by using both techniques). After the identification of norovirus as the putative agent, the physician involved discontinued active sample collection. Although the low amount of DNA amplified did not allow strain characterization by sequencing, norovirus was confirmed in 9 samples by RLBH with specific probes and genotyped as Birmingham (4 strains), Lordsdale (1), and Leeds (2). Two samples were not identified.

**Table 1:** Risk factors and food items associated with the presence of gastroenteritis in resort guests, case–control study, central Italy, June–September 2003*

Risk factor	Univariate statistical analysis				Multivariate logistic regression	
	OR	95% CI	χ^2^	p value	Coefficients	95% CI
Sea bathing	6.62	2.92–15.00	24.51	<0.01	4.76	1.99–11.42
Use of toilets and showers in cabins and chalet	3.40	1.95–5.90	19.53	<0.01	3.44	1.89–6.24
Use of cabin and villa showers supplied with drinking water	3.11	1.78–5.42	16.52	<0.01	–	–
Use of shared shower facilities supplied with nondrinking water	2.96	1.68–5.20	14.77	<0.01	2.49	1.32–4.68
Use of non–drinking water for various purposes (e.g., laundry, washing dishes, oral hygiene)	2.63	1.46–4.72	10.82	<0.01	–	–
Use of drinking water distributed to cabins and villas	2.15	1.24–3.72	7.54	<0.01	–	–
Use of swimming pools	2.11	1.21–3.69	6.99	<0.01	–	–
Use of bottled drinking water	0.36	0.09–1.39	2.39	>0.05	–	–
River bathing	3.05	0.31–29.81	1.02	>0.05	–	–
Use of common toilets	1.38	0.81–2.33	1.44	>0.05	–	–
Use of drinking water collected at hygienic services (e.g., bathrooms, showers, sinks) and fountains	1.55	0.77–3.11	1.51	>0.05	–	–
Use of mineral water for cooking	1.63	0.61–4.37	0.96	>0.05	–	–
Use of ice	0.79	0.44–1.44	0.57	>0.05	–	–
Use of drinking water collected at hygienic services (e.g., bathrooms, showers, sinks) and fountains for cooking	1.22	0.70–2.11	0.49	>0.05	–	–
Use of drinking water collected at permanent facilities (cabins and villas)	1.19	0.52–2.69	0.17	>0.05	–	–
Water massage at swimming pool	0.85	0.28–2.61	0.08	>0.05	–	–
Consumption of food items						
Sterile canned food	6.18	0.04–862.34	3.54	>0.05	–	–
Cooked vegetables	2.82	0.23–34.85	3.70	>0.05	–	–
Cooked eggs and egg preparations	1.97	0.04–106.07	0.61	>0.05	–	–
Pasta and cooked cereals	1.73	0.32–9.48	2.15	>0.05	–	–
Salami	1.18	0.14–9.68	0.13	>0.05	–	–
Milk and dairy products	1.00	0.2–5.04	0.00	>0.05	–	–
Cooked meat preparations	0.76	0.16–3.57	0.62	>0.05	–	–
Pizza, sandwiches, etc.	0.49	0.1–2.53	3.88	>0.05	–	–
Salads, fruits, raw vegetables	0.29	0.06–1.47	12.22	>0.05	–	–
Cooked fish preparations	0.25	0.03–1.93	10.20	>0.05	–	–
Croissants	0.18	0–26.86	2.97	>0.05	–	–

*Campylobacter* sp. was isolated in 1 of 14 samples tested, and 3 of 8 samples were positive for rotavirus. Results of all other microbiologic tests were negative.

In the environmental survey, we evaluated the layout and condition of the water pipelines supplying drinking water and the groundwater collected from 2 wells; these wells supplied water to showers, laundry facilities, public toilets, and irrigation pipelines. Water samples were then tested for fecal or pathogen contamination. We used the fluorescein test (*13*) to check for connections between systems conveying drinking water and non–drinking water within the resort. One hundred grams of sodium fluorescein were added to the 100-m^3^ tanks for non–drinking water (10^–3^ g/L), and passage of colored water to the drinking-water pipe system was monitored at 7 supply points by on-the-spot and laboratory spectrophotometric measurements. Eight activated carbon fluorescence traps were positioned for 4 days; absorbed fluorescein was then determined by spectrofluorimetry.

Table 2 shows the results of microbiologic tests conducted on the water samples. Results were assessed according to the reference limits established by Italian law for regulation of drinking water (*14*).

**Table 2:** Microbiologic analysis of water used within holiday resort, central Italy, case-control study, June–September 2003

Test	Results, positive/no. samples examined (%)		
	Drinking water	Non–drinking water*	Swimming pool water
Microbial count at 36°C*	4/49 (8)	8/20 (40)	0/7
Microbial count at 22°C†	4/49 (8)	9/20 (45)	0/7
*Clostridium perfringens‡*	0/49	5/20 (25)	Not tested
Total coliforms§	0/49	8/19 (42)	Not tested
*Escherichia coli*¶	0/49	0/20	0/7
Enterococcii#	2/49 (4)	7/20 (35)	0/7
Pseudomonas aeruginosa**	9/49 (18)	5/20 (25)	1/7 (14)
*Salmonella* spp.††	0/13	1/17 (6)	0/5
*Vibrio cholerae*‡‡	Not tested	0/9	Not tested
Coagulase-positive staphylococci (*S. aureus* and other species)§§	Not tested	Not tested	3/7 (43)
Norovirus antigen¶¶	Not tested	3/3 (100)	Not tested

*Limit of acceptability: drinking water = 100 CFU/mL; swimming pool water = <100 CFU/mL. †Limit of acceptability: drinking water = 100 CFU/mL; swimming pool water = <200 CFU/mL. ‡Limit of acceptability: drinking water = 0 CFU/mL. §Limit of acceptability: drinking water = 0 CFU/100 mL. ¶Limit of acceptability: drinking water = 0 CFU/mL; swimming pool water = 0 CFU/100 mL. #Limit of acceptability: drinking water = 0 CFU/100 mL; swimming pool water = 0 CFU/100 mL. **Limit of acceptability: drinking water = 0 CFU/100 mL; swimming pool water = <1 CFU/100 mL. ††Limit of acceptability: drinking water = absent/100 mL;.swimming pool water = absent/100 mL. ‡‡Limit of acceptability: drinking water = absent/100 mL. §§Limit of acceptability: swimming pool water = <1 CFU/100 mL. ¶¶Limit of acceptability: drinking water = sample volume examined.

Noroviruses were present in the 3 non–drinking-water samples and 2 of 3 sea samples examined, identified as either genotype Lordsdale or Leeds. The on-site fluorescein test detected fluorescein in 2 bungalows, and subsequent analysis of fluorescence traps showed that fluorescein was in a fountain supplied by the drinking-water system and in 2 other bungalows.

The environmental survey also included a study of the chemical pollution of groundwater wells near the resort and analyses of concentrations of ammonia, nitrites, and chloride by geographic distribution. Chloride, ammonia (Figure 1), and nitrite levels were statistically significantly higher in wells near the seashore and mouth of the river than levels in the inner wells. The opposite trend was shown for nitrates, which showed a decreasing concentration gradient from inland toward the sea, likely because of the difference in primary activities from agriculture to industry and tourism.

We proposed several prevention measures to eliminate connections and leaks between non–drinking-water and drinking-water systems and to ensure maintenance of pipelines that were put in place during 2004–2005. In 2004, 120 cases were reported to health services and all were related to the holiday resort; the number of cases decreased to 1 in 2005 and to 0 in 2006.

## Conclusions

Laboratory testing of stool samples from case-patients showed that norovirus, rotavirus, and *Campylobacter* spp. were possibly involved. However, the specific clinical signs, age of case-patients, and occurrence of secondary cases support our conclusion that norovirus was the most likely cause of the outbreak.

The rapid spread (approximately two thirds of case-patients had symptoms within 6 days after arrival) and the high rate of infection suggested a common source of infection, which caused the simultaneous exposure of a large number of resort guests. The twin peaks observed in the epidemic curve are likely related to guest turnover between July and August, when the lowest number of new cases was recorded. Persistence of the source probably caused the prolonged duration of the epidemics.

The case-control study identified sea bathing and use of shared toilet and shower facilities in cabins as statistically significant risk factors. Nygard et al. (*15*) have reported that use of shared shower facilities efficiently spreads infection. The observed fecal contamination in the sea and in the well water used to supply showers supports our conclusions. Tests conducted on well-water samples showed coliforms, enterococci, and *Salmonella togba*, which suggested specific fecal contamination.

The correlation between infection and use of cabin toilet and shower facilities may partly be due to contamination of these areas by infected persons, a theory that is supported by the high frequency of family clusters of infection. Fecal contamination of drinking-water systems may have arisen through connections with the non–drinking-water system in the resort or because of damaged pipework; fluorescein tracer was found in water from bungalow taps and drinking-water fountains.

The groundwater involved in contamination of both drinking-water and non–drinking-water systems was probably polluted by the river flowing into the sea nearby and by seawater infiltration; the concentration gradient of chemical indicators of organic pollution (ammonia) increased from the coast toward the inland area.

All well water samples and 2 seawater samples were positive for norovirus. Because 2 of the 3 genotypes (Lordsdale and Leeds) found in patients were also identified in these samples, we confirmed massive environmental contamination with human stools and the possible etiologic role of norovirus in this outbreak. Why the Birmingham genotype (most common in patient samples) was not found in the environmental samples is not clear, although the differential stability of viral strains in water and variation in the environmental shedding of different strains with time are possible explanations.

## References

[1] Páez Jiménez A, Pimentel R, Martínez de Aragón M, Hernández Pezzi G, Mateo Ontañon S, Martínez Navarro J. Waterborne outbreak among Spanish tourists in a holiday resort in the Dominican Republic, August 2002. Euro Surveill. 2004;9:21–3 [cited 22 Jan 2008]. Available from http://www.eurosurveillance.org/em/v09n03/0903-222.asp 10.2807/esm.09.03.00449-en 15075481

[2] Hafliger D, Hubner P, Luthy J. Outbreak of viral gastroenteritis due to sewage-contaminated drinking water. Int J Food Microbiol. 2000;54:123–6. 10.1016/S0168-1605(99)00176-2 10746582

[3] Kukkula M, Arstila P, Klossner ML, Maunula L, Bonsdorff CH, Jaatinen P. Waterborne outbreak of viral gastroenteritis. Scand J Infect Dis. 1997;29:415–8. 9360259 10.3109/00365549709011840

[4] Boccia D, Tozzi AE, Cotter B, Rizzo C, Russo T, Buttinelli G, Waterborne outbreak of Norwalk-like virus gastroenteritis at a tourist resort, Italy. Emerg Infect Dis. 2002;8:563–8. 12023910 10.3201/eid0806.010371 PMC2738487

[5] European Commission. Health and Consumer Protection Directorate General. Opinion of the Scientific Committee on Veterinary Measures Relating to Public Health on Norwalk-like viruses 2002 [cited 2007 Sept 20]. Available from http://europa.eu.int/comm/food/fs/sc/scv/out49_en.pdf

[6] Martin SW, Meek AH, Willeberg P. Veterinary epidemiology. Principles and methods. Ames (IA): Iowa State University Press; 1987. p. 121–48.

[7] Hosmer D, Lemeshow S. Applied logistic regression. New York: Wiley & Sons; 1989.

[8] Brinkler JP, Blacklow NR, Estes MK, Moe CL, Schwab KJ, Herrmann JE. Detection of Norwalk virus and other genogroup 1 human caliciviruses by a monoclonal antibody, recombinant-antigen-based immunoglobulin M capture enzyme immunoassay. J Clin Microbiol. 1998;36:1064–9. 9542938 10.1128/jcm.36.4.1064-1069.1998 PMC104690

[9] Hale AD, Tanaka TN, Kitamoto N, Ciarlet M, Jiang X, Takeda N, Identification of an epitope common to genogroup 1 “Norwalk-like viruses.” J Clin Microbiol. 2000;38:1656–60. 10747162 10.1128/jcm.38.4.1656-1660.2000 PMC86516

[10] Herrmann JE, Blacklow NR, Perron DM, Cukor G, Krause PJ, Hyams JS, Monoclonal antibody enzyme immunoassay for detection of rotavirus in stool specimens. J Infect Dis. 1985;152:830–2. 2995504 10.1093/infdis/152.4.830

[11] Cukor G, Perron DM, Hudson R, Blacklow NR. Detection of rotavirus in human stools by using monoclonal antibody. J Clin Microbiol. 1984;19:888–92. 6088573 10.1128/jcm.19.6.888-892.1984 PMC271205

[12] Vinjé J, Koopmans MP. Simultaneous detection and genotyping of “Norwalk-like viruses” by oligonucleotide array in a reverse line blot hybridization format. J Clin Microbiol. 2000;38:2595–601. 10878050 10.1128/jcm.38.7.2595-2601.2000 PMC86977

[13] US Environmental Protection Agency. Draft manual of practice identification of illicit connections. 1990 [cited 2007 Sep 20]. Available from http://www.p2pays.org/ref/17/16143.pdf

[14] Italian Decree n. 31, dated 2 Feb 2001. Transposition of European Directive 98/83/EC on water quality for human consumption. Official Journal n. 52, 2001 Mar 3.

[15] Nygard K, Vold L, Halvorsen E, Bringeland E, Rottingen JA, Aavitsland P. Waterborne outbreak of gastroenteritis in a religious summer camp in Norway, 2002. Epidemiol Infect. 2004;132:223–9. 10.1017/S0950268803001894 15061496 PMC2870097

